# Baseline mood and non-motor symptom burden are associated with cognitive progression in Parkinson’s disease: an interpretable follow-up cohort analysis with separate neuroimaging, molecular, and digital analyses in independent samples

**DOI:** 10.3389/frai.2026.1924525

**Published:** 2026-09-07

**Authors:** Jiangbin Ren, Jianghao Ren, Haoheng Yu, Hong Gao, Kan Wang, Chengjie Xu

**Affiliations:** 1 Department of Neurosurgery, Huanhu Hospital Affiliated to Tianjin Medical University, Tianjin Medical University, Tianjin, China; 2 Department of General Surgery, Zhejiang University School of Medicine Sir Run Run Shaw Hospital, Hangzhou, China; 3 Department of Neurology, Tongji Hospital, Tongji Medical College, Huazhong University of Science and Technology, Wuhan, China; 4 Department of Acupuncture and Moxibustion, Shanxi Hospital of Acupuncture and Moxibustion, Shanxi University of Chinese Medicine, Taiyuan, China; 5 Department of Neurosurgery, The Fourth Affiliated Hospital of Harbin Medical University, Harbin, China

**Keywords:** cognitive progression, digital phenotyping, interpretable machine learning, multimodal biomarkers, Parkinson’s disease, real-world data, resting-state fMRI

## Abstract

Clinically useful artificial intelligence and machine-learning studies in healthcare require interpretable features, internal validation, and explicit boundaries between primary inference and external context. Among 1,612 baseline Parkinson’s Progression Markers Initiative (PPMI) participants, 1,439 had evaluable post-baseline cognition and contributed 5,909 records through Year 5. Composite cognitive progression occurred in 720 participants (50.0%). Each standard deviation increase in baseline mood/non-motor burden was associated with higher odds of progression (adjusted odds ratio 1.37, 95% confidence interval 1.20–1.55). The 1,000-resample participant bootstrap interval was 1.20–1.57, and estimates were stable across 1- to 5-year windows (odds ratios 1.35–1.41). In 10 repetitions of stratified 5-fold cross-validation, adding composite burden to clinical covariates produced a modest increase in mean area under the receiver operating characteristic curve from 0.665 to 0.682. We interpreted the PPMI result alongside separate neuroimaging, transcriptomic, and digital analyses in other samples and specified a future same-participant study; the separate analyses were not used for participant-level integration or validation. In the small resting-state functional magnetic resonance imaging cohort, most static and dynamic comparisons did not survive false-discovery-rate correction; two threshold-specific network-based-statistic components were retained as exploratory hypotheses. Molecular rankings were consistent with previously reported Parkinson’s disease biology, while wearable and voice datasets demonstrated feasibility for the source-task only. Baseline mood/non-motor assessment may support future risk-enrichment research, but the limited cross-validated increment, absence of external clinical validation, and lack of participant-matched multimodal data preclude clinical implementation.

## 1 Introduction

Parkinson’s disease (PD) is a growing public health challenge and a clinically heterogeneous neurodegenerative disorder in which motor symptoms are only one component of disability (Dorsey et al., 2018). Contemporary descriptions emphasize cognitive, psychiatric, autonomic, sleep, and sensory symptoms as integral to disease burden rather than secondary features (Bloem et al., 2021; Poewe et al., 2017). Cognitive impairment is especially consequential because it affects independence, caregiver burden, and trial design, yet prediction near diagnosis remains imprecise even though dementia becomes common with longer disease duration (Aarsland et al., 2021; Hely et al., 2008). This combination of high clinical impact and limited early prediction accuracy makes PD an instructive test case for artificial intelligence (AI) and real-world data approaches.

AI and machine learning are increasingly used to model disease risk, prognosis, and treatment response from complex health data (Rajkomar et al., 2019). In neurodegenerative disease, however, clinically useful AI requires more than high aggregate accuracy. Models must be interpretable, robust to dataset artifacts, transparent in reporting, and connected to plausible disease biology (Collins et al., 2015; Rudin, 2019; Norgeot et al., 2020). This is particularly important for predicting cognitive progression in PD, where symptoms, neuroimaging, molecular pathways, and digital behaviors may provide complementary but non-identical views of vulnerability. A practical AI-ready workflow should therefore distinguish the primary inferential dataset from analyses in other samples used for biological context and planning external validation.

Mood and non-motor symptoms are attractive real-world risk features because they are common, scalable, and routinely assessed. Depression, anxiety, sleepiness, autonomic symptoms, apathy, fatigue, and impulse control-related symptoms may reflect distributed neurotransmitter and network vulnerability rather than isolated comorbidity (Chaudhuri and Schapira, 2009; Schapira et al., 2017). Their early occurrence also supports the idea that symptom burden can mark broader multisystem risk before overt cognitive decline is established (Pont-Sunyer et al., 2015). For an interpretable risk model, this creates an opportunity: simple clinical features can be tested against subsequent outcomes and interpreted alongside independent imaging, digital, and molecular resources to generate hypotheses for later participant-matched studies.

The Parkinson’s Progression Markers Initiative (PPMI) provides a suitable observational resource for this question because it combines standardized baseline symptom assessments with repeated cognitive follow-up (Parkinson Progression Marker Initiative, 2011). Resting-state functional magnetic resonance imaging (fMRI) can provide independent brain-network context by characterizing distributed functional organization (Biswal et al., 1995; Fox and Raichle, 2007). Transcriptomic, pathway, and digital phenotype resources can separately indicate whether hypotheses arising from the clinical analysis are compatible with prior biological knowledge or feasible monitoring approaches. Such a staged framework is aligned with current AI-in-healthcare priorities: heterogeneous health data should generate clinically useful, auditable, and biologically interpretable evidence rather than opaque prediction scores alone (Topol, 2019).

The Frontiers in Artificial Intelligence context also motivates a careful definition of AI readiness. The contribution is not the novelty of logistic regression itself, nor a black-box classifier presented as a finished clinical device. It is an auditable modeling workflow that combines transparent feature construction, adjusted association models, unsupervised symptom phenotyping, participant-level bootstrapping, repeated cross-validation, explicit missing-follow-up eligibility, and conservative separation of analyses conducted in different samples. These properties address recurring AI-in-healthcare problems of leakage, optimism, opacity, and misleading cross-dataset validation. Network analysis, digital benchmarking, and molecular prioritization were performed in separate datasets only to inform future participant-level model design.

**Figure 1:**
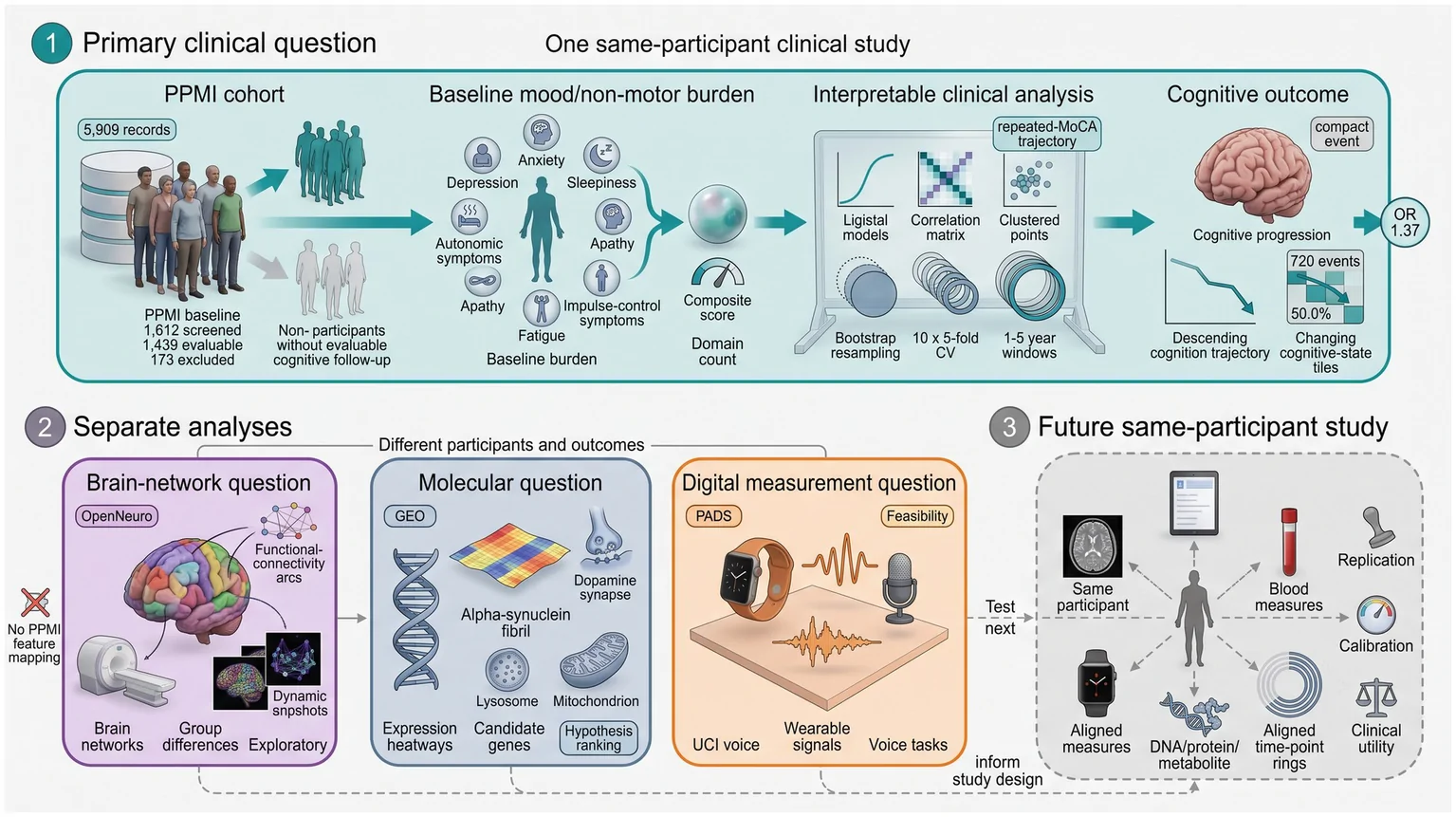
Primary PPMI analysis, separate analyses, and future same-participant study.

In this study, we tested whether baseline mood/non-motor burden was associated with 0–5 year cognitive progression in PPMI. Figure 1 distinguishes three stages: the primary PPMI analysis, separate resting-state fMRI, transcriptomic, and digital analyses in other samples, and a future same-participant study. The primary inference comes only from the PPMI follow-up cohort. No participant-level multimodal integration was performed, no analysis in another sample validated the PPMI association, and no output from one current analysis served as input to another. The future stage specifies how aligned clinical, imaging, digital, and biospecimen measurements could be evaluated within the same participants; it is not presented as a result of the current study.

## 2 Materials and methods

### 2.1 Study design

This study distinguished three stages (Figure 1). First, the primary PPMI analysis tested whether baseline mood and non-motor symptom burden were associated with 0–5 year cognitive progression. Second, OpenNeuro resting-state fMRI, public transcriptomic and curated pathway resources, and digital phenotype datasets were analyzed separately within their own samples and outcomes to address exploratory network anatomy, biological hypotheses, and measurement feasibility, respectively. Third, available multi-omics resources were used only to specify a future same-participant study design. The current analyses were not connected analytically: the other datasets contained different participants, were not linked to PPMI records, did not validate the PPMI association, and did not transfer features from imaging to molecular or from molecular to digital analyses. Global Burden of Disease (GBD) 2023 estimates were retained only as population-level background context (Global Burden of Disease Collaborative Network, 2024).

### 2.2 PPMI clinical cohort

PPMI is an observational follow-up study designed to identify PD progression biomarkers (Parkinson Progression Marker Initiative, 2011). Baseline participants whose primary-diagnosis variable indicated PD (PRIMDIAG = 1) yielded 1,612 potential participants. A participant entered a window-specific risk set only if baseline cognition and at least one post-baseline Montreal Cognitive Assessment (MoCA) or PPMI cognitive-category observation were available within that window. The 5-year primary risk set therefore included 1,439 participants and 5,909 records; 173 baseline participants without evaluable cognitive follow-up were not classified as non-progressors. PD diagnostic context followed PPMI adjudication and contemporary Movement Disorder Society (MDS) clinical diagnostic principles (Postuma et al., 2015).

Baseline variables included age, sex, education, disease duration, MoCA, Movement Disorder Society-Unified Parkinson’s Disease Rating Scale (MDS-UPDRS) part III, Hoehn and Yahr stage, levodopa-equivalent daily dose (LEDD), and PD treatment status. MoCA was used as the primary global cognitive scale (Nasreddine et al., 2005). Depressive symptoms were measured with the Geriatric Depression Scale (GDS; Yesavage et al., 1982–1983). Anxiety was represented by the State–Trait Anxiety Inventory (STAI) measures (Spielberger et al., 1983). Daytime sleepiness was represented by the Epworth Sleepiness Scale (ESS; Johns, 1991). Autonomic burden was represented by the Scales for Outcomes in Parkinson’s Disease-Autonomic (SCOPA-AUT). Impulse control-related symptoms were represented by the Questionnaire for Impulsive-Compulsive Disorders in Parkinson’s Disease (QUIP; Weintraub et al., 2009). Motor severity was represented by MDS-UPDRS part III (Goetz et al., 2008). LEDD was summarized using published conversion principles (Tomlinson et al., 2010).

### 2.3 Exposure construction and cognitive outcomes

Primary exposures were GDS, STAI, ESS, SCOPA-AUT, MDS-UPDRS part I depressed mood, anxiety, apathy, and fatigue items, QUIP-any status, a composite mood/non-motor burden score, and a symptom-domain count. The composite burden score was calculated by z-scoring available symptom scales and averaging available z-scores before re-standardization. The symptom-domain count summed binary domain flags for elevated GDS, upper-quartile STAI, ESS ≥ 10, upper-quartile SCOPA-AUT, QUIP-any status, and non-zero MDS-UPDRS part I mood/non-motor items.

The primary outcome was composite 0–5 year cognitive progression, defined as any worsening of the PPMI cognitive-category variable from baseline or any MoCA decline of at least 2 points from baseline within the window. Five years was chosen as the primary horizon to capture clinically meaningful early-to-intermediate progression while retaining a large evaluable cohort across scheduled PPMI visits. Because a longer window permits more event accrual and may be more affected by attrition, the same outcome and adjustment set were re-estimated for 1-, 2-, 3-, and 4-year windows. Sensitivity outcomes included MoCA decline of at least 3 points, cognitive progression among participants with normal baseline cognition, and incident mild cognitive impairment (MCI) or dementia. Interpretation followed established PD-MCI and PD dementia criteria (Litvan et al., 2012; Emre et al., 2007).

### 2.4 PPMI statistical analysis

Baseline characteristics were summarized by cognitive progression status using means with standard deviations (SDs) for continuous variables and counts for categorical variables. The *p*-values in Table 1 were based on two-sided Welch two-sample t-tests for continuous variables and Pearson chi-square tests for categorical variables; these tests were descriptive and unadjusted. Exposure distributions and exposure-outcome correlations were summarized using Spearman rank correlations. Fully adjusted logistic regression models estimated odds ratios (ORs) per SD of each baseline exposure with 95% confidence intervals (CIs) for composite cognitive progression and MoCA decline. Models adjusted for age, sex, education, disease duration, baseline MoCA, MDS-UPDRS part III, Hoehn and Yahr stage, LEDD, and PD treatment status when these variables were available.

**Table 1:** Baseline characteristics of the 1,439-participant PPMI analytic cohort with evaluable cognitive follow-up, by 0–5-year composite cognitive progression status.

Variable	No progression	Progression	*p*-value	Tests
Age, years	61.49 (9.60)	64.50 (9.56)	2.96e-09	Welch two-sample t-test
Education, years	15.95 (2.82)	15.61 (3.36)	0.037	Welch two-sample t-test
Disease duration, years	1.21 (1.72)	1.31 (1.67)	0.292	Welch two-sample t-test
MoCA score	26.53 (2.56)	26.91 (2.77)	0.007	Welch two-sample t-test
MDS-UPDRS part III	21.00 (9.48)	23.05 (10.68)	1.87e-04	Welch two-sample t-test
Hoehn and Yahr stage	1.63 (0.51)	1.69 (0.52)	0.021	Welch two-sample t-test
LEDD, mg/day	76.71 (233.82)	138.73 (315.22)	2.40e-05	Welch two-sample t-test
GDS	2.17 (2.55)	2.93 (2.98)	2.76e-07	Welch two-sample t-test
STAI	63.14 (17.61)	67.72 (19.63)	3.63e-06	Welch two-sample t-test
ESS	5.55 (3.81)	6.25 (3.99)	7.83e-04	Welch two-sample t-test
SCOPA-AUT	10.08 (6.65)	11.88 (7.37)	1.48e-06	Welch two-sample t-test
Composite mood/non-motor burden, z score	−0.17 (0.92)	0.14 (1.03)	2.33e-09	Welch two-sample t-test
Sex	Female: 282; Male: 437	Female: 270; Male: 450	0.537	Pearson chi-square test
PD treatment status	No: 632; Yes: 86	No: 564; Yes: 156	1.30e-06	Pearson chi-square test
QUIP-any status	No: 570; Yes: 144	No: 530; Yes: 188	0.008	Pearson chi-square test

*p*-values are two-sided Welch two-sample t-tests for continuous variables and Pearson chi-square tests for categorical variables; they are descriptive and unadjusted.

Repeated-measures MoCA models tested year-by-exposure interactions using participant-clustered linear models. Symptom clusters were generated from median-imputed, standardized symptom measures using principal component analysis (PCA) for visualization and k-means clustering with three clusters. Internal stability of each adjusted exposure association was assessed with 1,000 non-parametric participant bootstrap resamples and percentile intervals. Prediction benchmarks used 10 repetitions of stratified 5-fold cross-validation with all imputation and scaling confined to each training fold. Regularized logistic models compared clinical covariates alone, clinical covariates plus composite burden, and clinical covariates plus the individual symptom measures; area under the receiver operating characteristic curve (AUC) and Brier score were recorded. False-discovery-rate (FDR) correction used the Benjamini–Hochberg procedure (Benjamini and Hochberg, 1995).

### 2.5 OpenNeuro resting-state fMRI cohort and preprocessing

**Figure 2:**
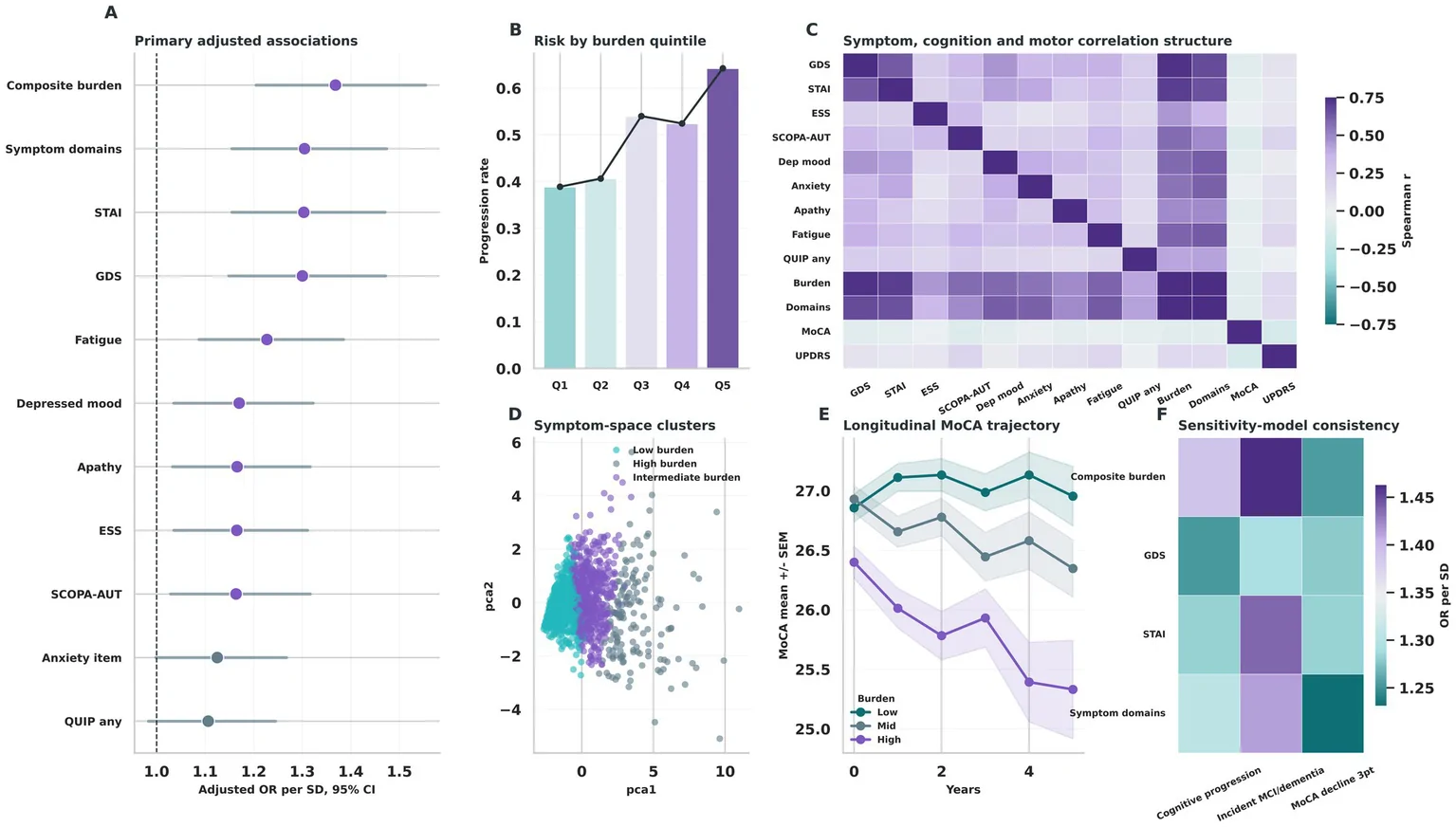
PPMI clinical phenotype and cognitive progression during follow-up. **(A)** Fully adjusted logistic associations between baseline mood/non-motor exposures and composite cognitive progression. **(B)** Cognitive progression rates across mood/non-motor burden quintiles. **(C)** Baseline symptom-cognition-motor correlation structure. **(D)** Symptom-space clusters. **(E)** Repeated MoCA trajectories by baseline burden group. **(F)** Sensitivity-model consistency across alternative cognitive outcomes and covariate specifications.

The independent resting-state fMRI dataset was OpenNeuro ds005892, which contains healthy controls, PD with normal cognition (PD-NC), and PD with mild cognitive impairment (PD-MCI; Kemp et al., 2025). The analyzed imaging cohort included 55 participants with complete fMRIPrep-derived data: 22 controls, 18 PD-NC participants, and 15 PD-MCI participants. The dataset did not contain PPMI-aligned composite burden, STAI, GDS, or related exposure variables; therefore, we did not map the Figure 2 clinical features into brain-network space. Resting-state fMRI was used only for independent group-level network context. Resting-state functional connectivity was motivated by established intrinsic blood-oxygen-level-dependent (BOLD) connectivity principles (Biswal et al., 1995; Fox and Raichle, 2007).

Preprocessed fMRI outputs were generated with fMRIPrep (Esteban et al., 2019). Schaefer et al.’s 100-parcel, 7-network atlas in Montreal Neurological Institute (MNI) space was used for region-of-interest (ROI) extraction (Schaefer et al., 2018). Yeo 7-network assignments provided the functional-network family labels (Yeo et al., 2011). ROI time series were extracted with nilearn NiftiLabelsMasker (Abraham et al., 2014). Denoising included fMRIPrep motion parameters, white matter, cerebrospinal fluid, global-signal terms, anatomical component-based noise correction (aCompCor), cosine drifts, and one-hot outlier regressors when available. Motion summaries were retained because head motion can induce spurious functional-connectivity group differences (Behzadi et al., 2007; Power et al., 2012; Satterthwaite et al., 2012).

### 2.6 Static and dynamic connectivity analysis

ROI time series were detrended, standardized, and filtered from 0.01 to 0.1 Hz before connectivity estimation. Static connectivity matrices were generated as Pearson correlations among Schaefer100 ROI time series and Fisher-z transformed for analysis. Subject-level features included global connectivity summaries, within-network connectivity, between-network connectivity, motion summaries, and edge-level Fisher-z values. Group tests used Kruskal–Wallis tests across controls, PD-NC, and PD-MCI participants, followed by Benjamini–Hochberg FDR correction.

Dynamic connectivity was assessed using sliding-window, coactivation-pattern (CAP), and Hilbert phase-synchrony representations. Sliding-window analysis used 30 repetition-time (TR) correlation windows and k-means clustering into four states (Allen et al., 2014). Dynamic connectivity interpretation followed recommendations to treat time-varying metrics cautiously in small cohorts (Hutchison et al., 2013). CAP analysis selected the top 20% high-root-mean-square (RMS) frames per subject with a minimum of 20 frames and clustered coactivation patterns into four states (Liu and Duyn, 2013). Hilbert phase-synchrony analysis estimated instantaneous phase-locking summaries and clustered them into four states (Lachaux et al., 1999).

### 2.7 Network-based statistics and hemispheric summaries

Permutation network-based statistics (NBS) tested whether edge-level group differences formed connected components across Schaefer100 ROI pairs. The edge statistic was the omnibus Kruskal–Wallis H statistic across control, PD-NC, and PD-MCI groups. Primary component correction used maximum connected-component extent under 5,000 group-label permutations. Secondary correction used component intensity based on summed excess H above the primary threshold (Zalesky et al., 2010; Nichols and Holmes, 2002).

Hemispheric summaries included homotopic connectivity by network and interhemispheric/intrahemispheric contrasts. Network-pair concentration summarized the distribution of nominally stronger edge-level signals across Yeo network pairs. These analyses were used to interpret component-level organization and were not treated as independent confirmatory tests.

### 2.8 Transcriptomic, pathway, and candidate-hypothesis contextualization

Public Gene Expression Omnibus (GEO) series matrix files were analyzed to provide independent molecular context (Edgar et al., 2002; Barrett et al., 2013). Differential-expression outputs were generated from available processed matrices, while raw-only datasets were inventoried but not re-normalized. Curated pathway modules covered dopamine/synaptic signaling, lysosome-autophagy, mitochondrial biology, and neuroimmune-relevant biology. Pathway interpretation was informed by gene-set enrichment principles (Subramanian et al., 2005), and the Kyoto Encyclopedia of Genes and Genomes (KEGG) provided canonical pathway context (Kanehisa and Goto, 2000).

An exploratory network-context score summarized the concentration of imaging signals and was displayed alongside pathway and candidate-gene rankings. Candidate rankings combined prespecified PD, mood, cognition, and tractability priors with GEO differential-expression summaries and pathway overlap. Because this procedure used curated priors and measurements from unrelated participants, it was treated as descriptive hypothesis ranking rather than a statistical link from the PPMI phenotype to any molecular mechanism. Blood-expression results were interpreted in light of prior work showing that peripheral gene expression can capture molecular signals related to early PD (Scherzer et al., 2007), without assuming that peripheral expression directly measures the corresponding brain process.

### 2.9 Digital phenotype context and prospective multi-omics study design

Parkinson’s Disease Smartwatch (PADS) data were used to evaluate wearable movement associations with non-motor symptom summaries (Varghese et al., 2024). Open digital datasets included University of California, Irvine (UCI) voice and telemonitoring resources, which were treated as cross-sectional digital phenotype context. Smartphone-based PD symptom monitoring was included as background for scalable digital assessment (Arora et al., 2015). Telemonitoring voice analyses were interpreted with reference to prior speech-based progression modeling (Tsanas et al., 2010; Little et al., 2009; Bot et al., 2016). No molecular, pathway, or candidate-gene score was used as an input to any digital analysis.

Prospective multi-omics study planning was summarized across source availability, candidate hypotheses, and cell-type/pathway assay options. Candidate cell-type axes included dopaminergic neurons, gamma-aminobutyric acid (GABA)-ergic neurons, astrocytes, microglia, and immune-enriched compartments. Lysosome-autophagy hypotheses were interpreted against published lysosomal impairment in PD (Dehay et al., 2013), mitochondrial hypotheses against published mitochondrial dysfunction (Bose and Beal, 2016), and neuroimmune hypotheses against published neuroinflammation (Hirsch and Hunot, 2009). Alpha-synuclein-related hypotheses were interpreted against established Lewy pathology biology (Goedert et al., 2013). These scores describe a future validation design; they are not results from single-cell, spatial, or proteomic measurements in the analyzed PPMI participants.

### 2.10 Software and reproducibility

Analyses were implemented in Python using tabular, statistical, imaging, and machine-learning libraries. The scikit-learn module was used for imputation, scaling, k-means clustering, regularized logistic regression, random forests, and cross-validation (Pedregosa et al., 2011). The statsmodels module was used for regression modeling and multiple-testing utilities where applicable (Seabold and Perktold, 2010). The fMRI workflow used nilearn for atlas-based signal extraction and cached masking operations (Abraham et al., 2014). The reviewer-specific eligibility, bootstrap, repeated-cross-validation, and window analyses are reproduced by scripts/make_reviewer2_ppmi_revision.py in the submission package.

## 3 Results

### 3.1 Baseline mood and non-motor burden were associated with cognitive progression in PPMI

Of the 1,612 baseline participants with PD, 1,439 had an evaluable post-baseline MoCA or cognitive-category observation and contributed 5,909 records through Year 5. Composite cognitive progression occurred in 720 participants (50.0%). The 173 participants without evaluable cognitive follow-up were not assigned to the non-progression group. Baseline characteristics and exposure distributions are summarized in Tables 1, 2. Population-level GBD context is provided in Supplementary Figure S1 and Supplementary Tables 1–3, while cohort construction, variable coverage, and missingness are reported in Supplementary Tables 4–6 and Supplementary Figure S2.

**Table 2:** Baseline exposure distributions and Spearman correlations with composite cognitive progression.

Variable	*n*	Mean	SD	Median	q25	q75	Progression Spearman’s r	Progression Spearman’s *p*
GDS	1,432	2.554	2.798	2	0	4	0.146	2.73e-08
STAI	1,431	65.437	18.788	62	50	77	0.114	1.43e-05
ESS	1,433	5.901	3.911	5	3	8	0.095	3.22e-04
SCOPA-AUT	1,420	10.984	7.078	10	6	14	0.134	4.35e-07
MDS-UPDRS part I depressed mood	1,437	0.377	0.689	0	0	1	0.079	0.003
MDS-UPDRS part I anxiety	1,437	0.537	0.753	0	0	1	0.039	0.137
MDS-UPDRS part I apathy	1,437	0.278	0.62	0	0	0	0.069	0.009
MDS-UPDRS part I fatigue	1,437	0.742	0.867	1	0	1	0.109	3.36e-05
QUIP-any status	1,432	0.232	0.422	0	0	0	0.071	0.007
Composite mood/non-motor burden, z score	1,439	−0.011	0.991	−0.2	−0.714	0.428	0.173	4.22e-11
Symptom-domain count	1,439	2.499	2.13	2	1	4	0.135	2.97e-07
Composite cognitive progression	1,439	0.5	0.5					
MoCA decline > = 2 points	1,434	0.426	0.495					
MoCA decline > = 3 points	1,434	0.282	0.45					
Cognitive-category worsening	1,091	0.217	0.413					
Incident MCI or dementia	1,091	0.205	0.404					

The final five rows are binary cognitive-outcome summaries included for context; median, quartile, and progression-correlation cells are intentionally left blank because those statistics were calculated for baseline exposure variables only, not for the outcome variables themselves.

Baseline mood/non-motor burden was the clearest clinical signal. In fully adjusted complete-case models, each SD increase in the composite burden score was associated with higher odds of cognitive progression (OR 1.37, 95% CI 1.20–1.55; FDR-adjusted *p* = 3.63 × 10^-5; *n* = 1,298; Figure 2A). A symptom-domain count showed a similar association (OR 1.30, 95% CI 1.15–1.47; FDR-adjusted *p* = 1.66 × 10^-4). GDS, STAI, ESS, SCOPA-AUT, depressed mood, apathy, and fatigue associations also survived FDR correction; the MDS-UPDRS anxiety item and QUIP-any estimate were positive but did not survive correction. These estimates describe separately fitted exposure models and do not identify mutually independent symptom effects.

The association was also visible at the phenotype level. Cognitive progression increased from 38.9% in the lowest burden quintile to 64.2% in the highest quintile, although the middle quintiles were not strictly monotonic (Figure 2B). Baseline symptom, cognitive, and motor variables formed a correlated clinical space (Figure 2C), and unsupervised symptom-space clustering separated low-, intermediate-, and high-burden groups with progression rates of 41.8, 56.0, and 65.5%, respectively (Figure 2D). Participants with higher baseline burden also had lower repeated MoCA trajectories (Figure 2E), with the clearest year-by-exposure interactions for GDS and STAI. Alternative cognitive outcomes were directionally concordant (Figure 2F; Supplementary Tables 7–10; Supplementary Figures S3, S4).

Internal validation supported coefficient stability but only modest incremental discrimination. All 11 exposure models completed 1,000 of 1,000 bootstrap resamples; the composite-burden bootstrap 95% interval was 1.20–1.57. Across 1-, 2-, 3-, 4-, and 5-year risk windows, evaluable samples increased from 1,376 to 1,439 and event rates from 29.7 to 50.0%, while the adjusted composite-burden OR remained between 1.35 and 1.41. In 10× 5-fold cross-validation, mean AUC was 0.665 (SD 0.034) for clinical covariates, 0.682 (SD 0.030) after adding composite burden, and 0.673 (SD 0.028) after adding individual symptoms. Thus, burden added 0.017 AUC on average, a small internal increment rather than evidence of clinical utility. Full bootstrap, window, and fold-level results are reported in Supplementary Tables 68–70 and Supplementary Figure S19.

### 3.2 Resting-state fMRI in another sample provided exploratory brain-network context

We next examined a separate OpenNeuro resting-state fMRI sample containing controls, PD without cognitive impairment, and PD with mild cognitive impairment. This was not a mapping of the Figure 2 features: the imaging participants had no aligned composite burden, STAI, GDS, or related PPMI exposure measures. Motion and global-connectivity summaries were inspected as quality-control measures. Because these participants were not the same individuals as the PPMI cohort, all imaging analyses were interpreted as exploratory group-level anatomical context rather than participant-level or external validation.

**Figure 3:**
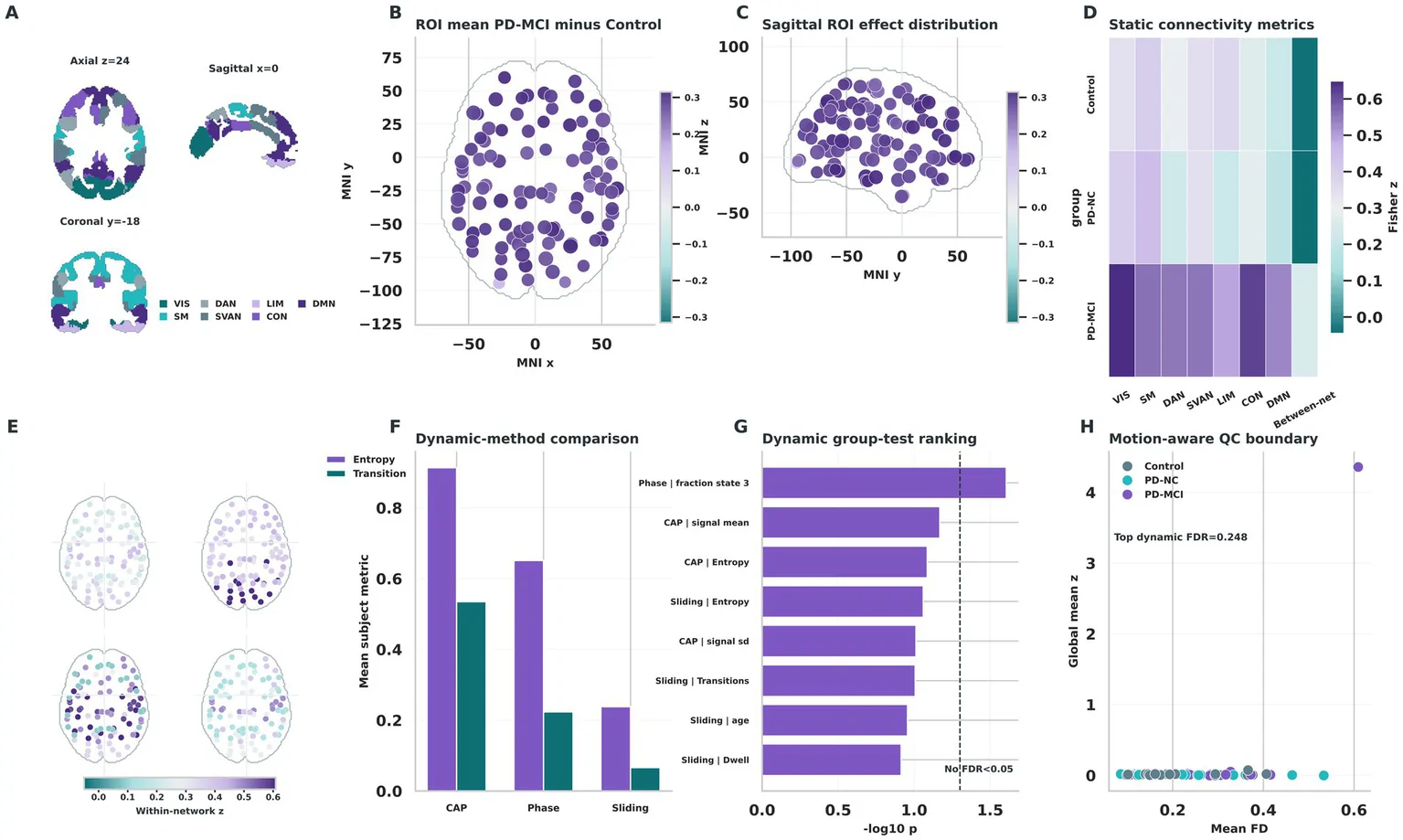
Exploratory static and dynamic resting-state brain-network context from the independent OpenNeuro cohort. **(A)** Schaefer/Yeo atlas slices with network-color labels. **(B)** Axial ROI-level mean PD-MCI-minus-control connectivity differences projected onto MNI brain coordinates. **(C)** Sagittal ROI-level PD-MCI-minus-control effect distribution. **(D)** Static network connectivity metrics by group. **(E)** Sliding-window dynamic state profiles projected onto Schaefer/Yeo brain-network coordinates after high-global-connectivity quality control. **(F)** Dynamic method comparison across sliding-window, CAP, and phase-synchrony representations. **(G)** Cross-method dynamic group-test ranking. **(H)** Motion and global-connectivity diagnostic plot. Most group comparisons did not survive FDR correction; panels are descriptive and do not validate the PPMI phenotype.

Static connectivity summaries did not yield a broad FDR-corrected network-level group effect. ROI-level PD-MCI-minus-control differences were projected onto Schaefer/Yeo brain coordinates for descriptive visualization (Figures 3A–C). Somatomotor and control-network summaries had the smallest nominal *p*-values, but neither survived FDR correction (Figure 3D). Accordingly, these patterns are candidate locations for future study, not evidence of network involvement in the PPMI clinical association.

Dynamic connectivity analyses provided a complementary exploratory view of time-varying network organization. Sliding-window, CAP, and phase-synchrony representations produced four-state solutions that could be visualized on the same network coordinate system (Figures 3E,F). No dynamic metric survived FDR correction across groups; the smallest nominal *p*-value involved a phase-synchrony state fraction (Figure 3G). Motion and global-connectivity diagnostics are shown to aid interpretation, but they do not convert the null-corrected comparisons into confirmatory evidence (Figure 3H). Full static and dynamic imaging outputs are provided in Supplementary Tables 11–26 and Supplementary Figures S5, S6.

### 3.3 Exploratory permutation NBS yielded threshold-dependent components in the OpenNeuro cohort

**Figure 4:**
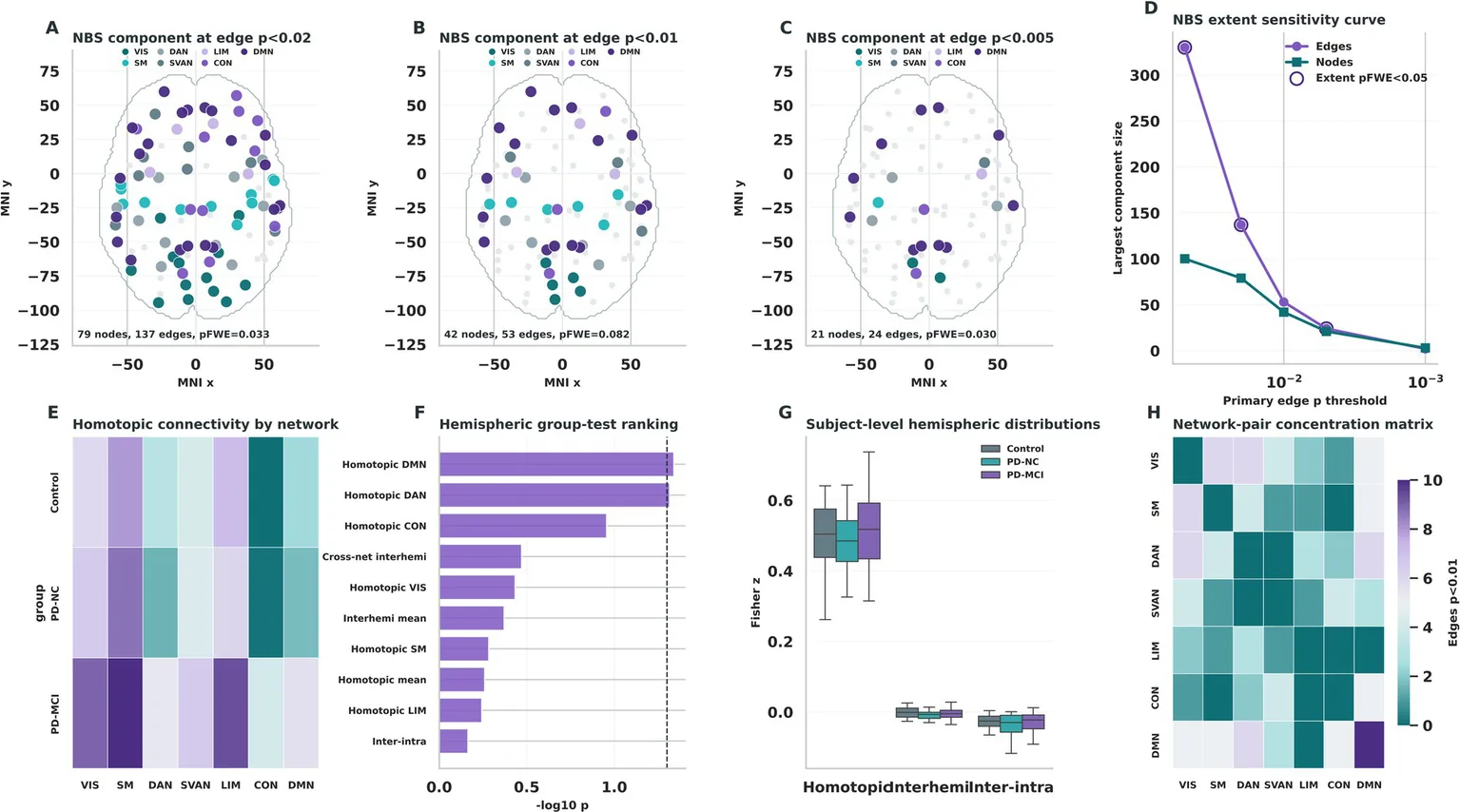
Exploratory permutation NBS and advanced connectivity summaries from the independent OpenNeuro cohort. **(A)** Permutation NBS component at primary edge threshold *p* < 0.02 projected onto Schaefer/Yeo ROI coordinates. **(B)** Component at *p* < 0.01. **(C)** Component at *p* < 0.005. **(D)** NBS component-extent sensitivity with FWE markers. **(E)** Homotopic connectivity by network. **(F)** Hemispheric metric group-test ranking. **(G)** Subject-level hemispheric distributions. **(H)** Descriptive network-pair counts for edges with uncorrected *p* < 0.01. Components at *p* < 0.02 and *p* < 0.005, but not *p* < 0.01, survived component-extent correction. Analyses were unadjusted for covariates, were not linked to PPMI participants, and should not be interpreted as validated biomarkers.

Permutation NBS was used to ask whether group differences within the OpenNeuro cohort formed connected components rather than scattered edges. At the *p* < 0.02 primary edge threshold, a 137-edge component survived extent correction (family-wise-error [FWE]-corrected *p* = 0.0330); at *p* < 0.005, a 24-edge component also survived correction (FWE-corrected *p* = 0.0298; Figures 4A–C). The intermediate *p* < 0.01 component did not survive correction, and the component fragmented as the threshold became more stringent (Figure 4D). This non-monotonic threshold sensitivity, the small cohort, and the absence of participant matching to PPMI limit the result to exploratory within-cohort component evidence.

The components were distributed across more than one anatomical system. Hemispheric and homotopic summaries showed nominal but not FDR-corrected effects (Figures 4E–G). A descriptive count of edges with uncorrected *p* < 0.01 was concentrated in Default-Default, Somatomotor-Visual, Dorsal Attention-Visual, Default-Dorsal Attention, and Default-Visual pairs (Figure 4H). These systems are reported only as hypotheses for future imaging analyses and were not used to infer a molecular mechanism. Full NBS, hemispheric, and network-pair outputs are reported in Supplementary Tables 27–33 and Supplementary Figures S7, S8.

### 3.4 Molecular analyses in other samples provided hypothesis-generating context

**Figure 5:**
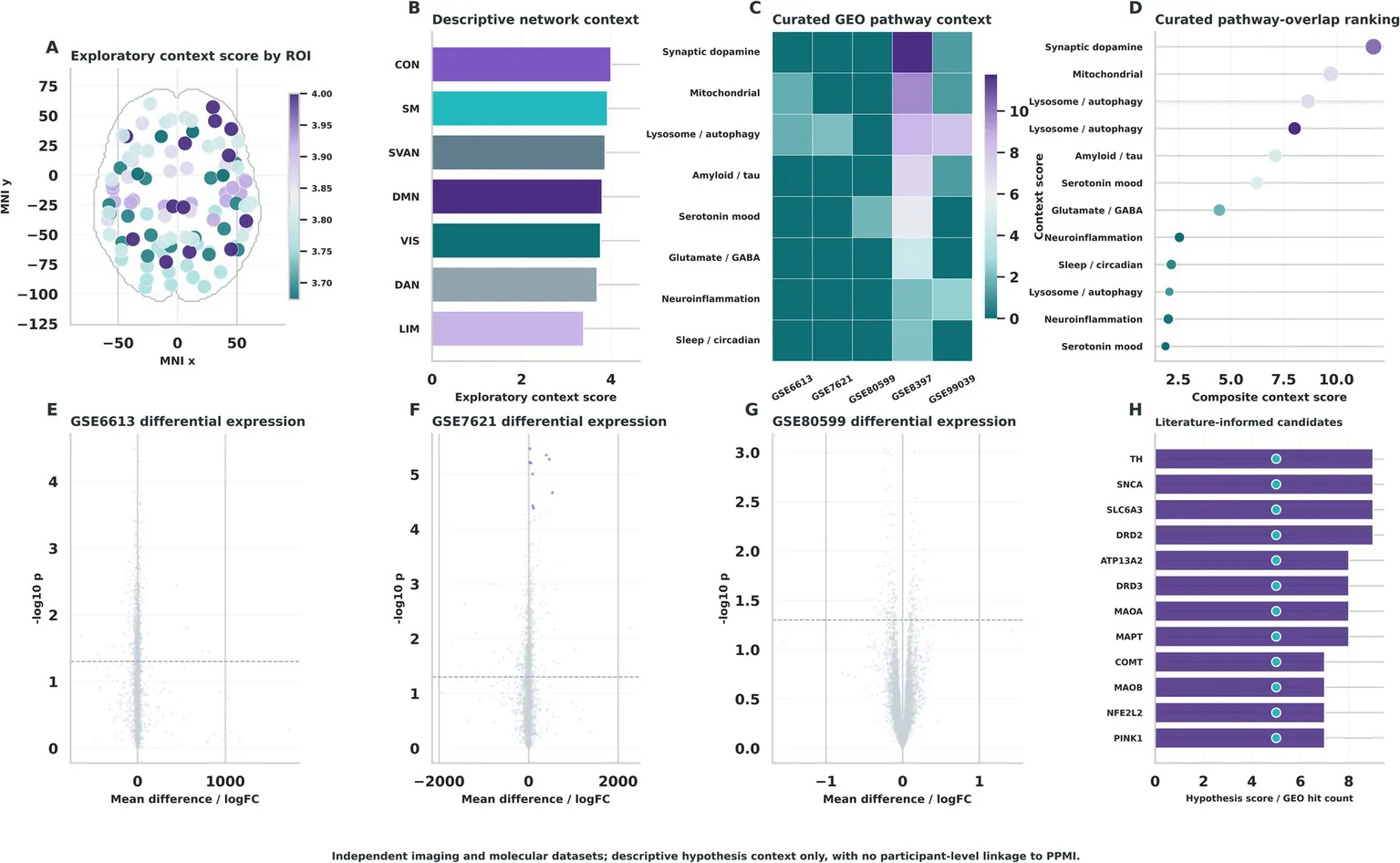
Independent imaging and molecular datasets used for hypothesis-generating context. **(A)** Exploratory network-context score projected to Schaefer/Yeo ROIs. **(B)** Descriptive network-context ranking. **(C)** Curated GEO pathway-context matrix across datasets and prespecified modules. **(D)** Pathway-overlap ranking, with point size reflecting overlap and color reflecting FDR within the source analysis. **(E)** GSE6613 differential-expression volcano plot. **(F)** GSE7621 volcano plot. **(G)** GSE80599 volcano plot. **(H)** Literature-informed candidate-gene ranking showing the composite hypothesis score and GEO hit count. Imaging and molecular measurements came from different participants; this figure does not demonstrate a brain-molecular link or validate mechanisms underlying the PPMI association.

Exploratory network-context scores were displayed alongside transcriptomic and curated pathway summaries from other sources (Figure 5). The descriptive scores were highest across control, somatomotor, salience/ventral-attention, default, visual, and dorsal-attention systems (Figures 5A,B). Because the scores summarize the imaging analyses above and are not based on paired imaging and molecular measurements, they neither demonstrate a brain-molecular link nor define validated imaging biomarkers.

Among the prespecified pathway modules, the largest overlap scores occurred for synaptic dopamine in GSE8397 and lysosome/autophagy in GSE99039, with other ranked results involving mitochondrial and lysosome/autophagy modules (Figures 5C,D). Differential-expression summaries across GEO datasets are shown in Figures 5E–G. A candidate-gene ranking that explicitly incorporated curated PD, mood, cognition, and tractability priors placed DRD2, SLC6A3, SNCA, and TH in the highest tier, followed by ATP13A2, DRD3, MAOA, and MAPT (Figure 5H). These rankings are consistent with biological systems described previously in PD literature, but the use of prespecified modules and priors precludes interpreting them as *de novo* mechanistic discoveries. They identify hypotheses for matched-biospecimen studies and do not provide molecular validation of the PPMI association. Supporting contextualization, differential-expression, pathway, and candidate-hypothesis outputs are reported in Supplementary Tables 34–39 and Supplementary Figures S9–S11.

### 3.5 Digital analyses in other samples addressed measurement feasibility

**Figure 6:**
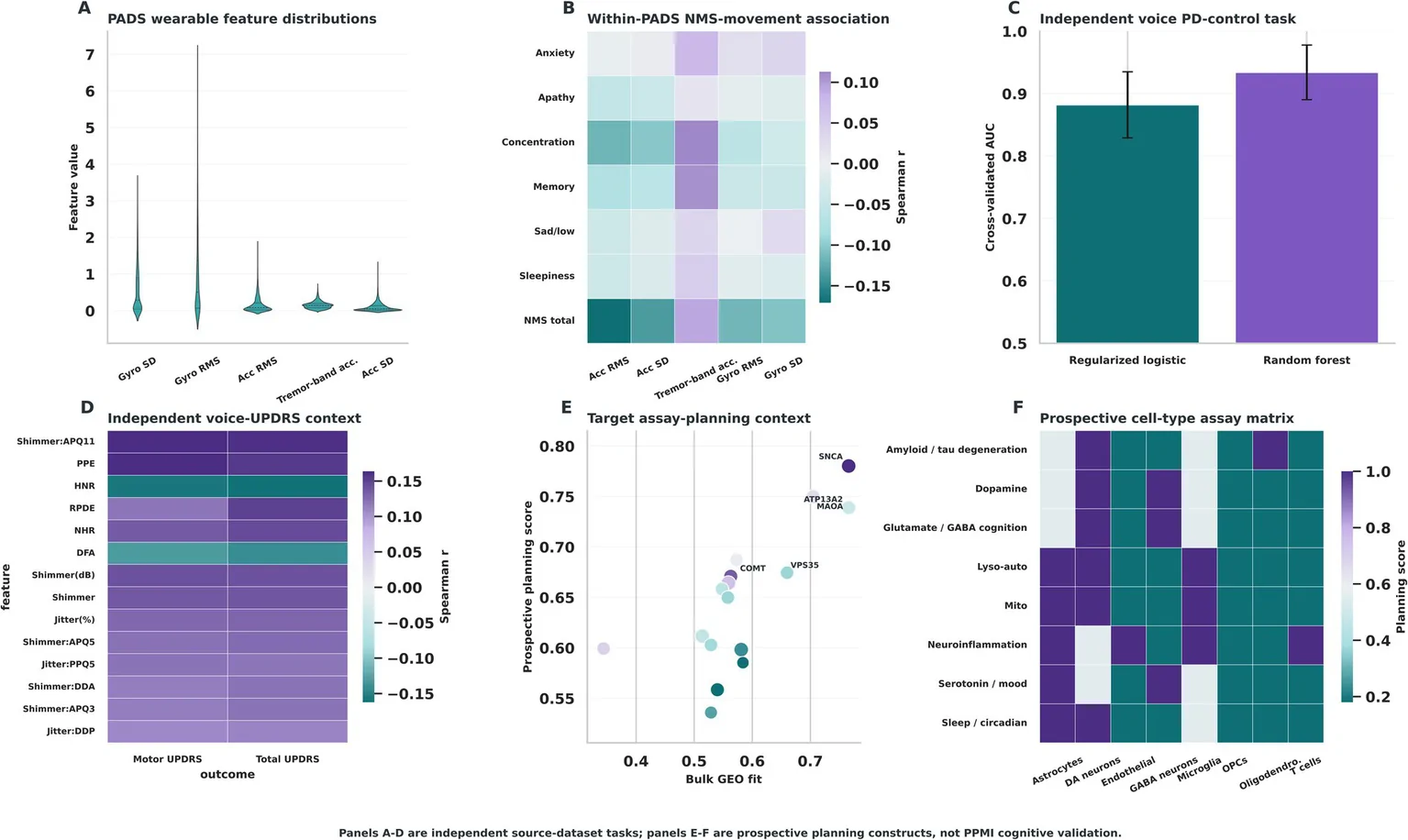
Independent digital context and prospective multi-omics assay planning. **(A)** PADS wearable feature distributions. **(B)** Non-motor symptom and movement-feature associations within PADS. **(C)** PD-versus-control benchmark in an independent open voice dataset. **(D)** Voice-feature correlations with motor and total UPDRS in an independent telemonitoring dataset. **(E)** Literature- and availability-informed target planning scores, with point size reflecting the candidate-hypothesis score and color reflecting proteomics fit. **(F)** Prospective cell-type/pathway assay matrix. Panels **(A–D)** address source-dataset tasks rather than PPMI cognitive progression; panels **(E,F)** are planning constructs, not completed molecular validation.

Digital phenotype datasets were analyzed separately as examples of scalable behavioral measurement in their own sampling frames. Their placement after the molecular section does not indicate a gene/pathway-to-digital analysis, and no molecular or imaging feature entered a digital model. In PADS, greater non-motor symptom burden was associated with lower wearable movement amplitude, most clearly for accelerometer RMS (Spearman r = −0.171; FDR-adjusted *p* = 0.0071; Figures 6A,B). In a separate open voice dataset, regularized logistic regression and random forest models distinguished PD from control speech recordings with mean AUCs of 0.882 and 0.934, respectively (Figure 6C). Telemonitoring voice features were associated with motor and total Unified Parkinson’s Disease Rating Scale (UPDRS) scores in another source dataset (Figure 6D). None of these datasets included the PPMI exposure-outcome pairing, so these analyses demonstrate source-task feasibility only and do not validate digital monitoring of cognitive progression.

The multi-omics planning exercise converted the candidate hypotheses into prospective assay options rather than analytical evidence. Planning scores were highest for SNCA, MAOA, ATP13A2, COMT, and VPS35 (Figure 6E). The cell-type/pathway matrix specified possible assays for dopamine-related modules in dopaminergic and GABAergic neurons, lysosome-autophagy and mitochondrial modules in astrocyte and microglial contexts, and neuroimmune modules in immune and microglial compartments (Figure 6F). These choices reflect literature-informed feasibility scores, not measured cell-type effects. Ancillary neuroimaging-resource summaries further separated analyses feasible with current data from modalities requiring prospective acquisition. Diffusion MRI connectomics, glymphatic DTI, and electrophysiology/source-localization analyses remain proposed experiments rather than completed evidence layers. Digital-context, prospective-assay, and modality-audit outputs are provided in Supplementary Tables 40–67 and Supplementary Figures S12–S18.

## 4 Discussion

This study found that baseline mood/non-motor burden was associated with 0–5 year cognitive progression in PD. The association was stable under participant bootstrap and across 1- to 5-year windows, but adding burden to clinical covariates improved repeated cross-validated AUC by only 0.017, and no external clinical cohort was available. The signal is therefore internally reproducible but not externally validated or ready for clinical prediction. Its AI relevance lies in the transparent construction and validation of accessible features and in the explicit handling of missing follow-up and heterogeneous external datasets, not in claiming algorithmic novelty for logistic regression. The findings remain associations and do not establish that mood or non-motor symptoms cause subsequent decline.

Several biological routes could plausibly produce this association, but none was directly tested here. Serotonergic raphe and noradrenergic locus-coeruleus degeneration, together with mesolimbic dopaminergic dysfunction, can affect mood, arousal, attention, and executive control; imaging and neuropathological studies support widespread monoaminergic involvement in PD rather than an exclusively nigrostriatal process (Remy et al., 2005; Politis and Niccolini, 2015; Schapira et al., 2017). Cholinergic basal-forebrain degeneration is also relevant because it has been associated with subsequent cognitive decline in *de novo* PD and may interact with frontostriatal deficits to reduce cognitive reserve (Ray et al., 2018; Halliday et al., 2014). Lewy pathology can extend from brainstem nuclei into limbic and neocortical territories, so a co-occurring cluster of affective, sleep, autonomic, and cognitive symptoms could reflect the spatial spread or combined expression of pathology across several neurotransmitter systems (Braak et al., 2003; Halliday et al., 2014). These mechanisms are not mutually exclusive, and the present clinical score cannot distinguish degeneration from medication effects, reactive mood symptoms, shared vascular or inflammatory factors, or early cognitive change influencing symptom reporting.

The independent imaging analyses provide only exploratory anatomical context. Most static, dynamic, hemispheric, and homotopic comparisons did not survive FDR correction. Although two threshold-specific NBS components survived component-extent correction, the intermediate threshold did not, and the small cohort was not matched to PPMI. The displayed default, attentional, salience, somatomotor, and visual patterns should therefore be treated as network hypotheses for a future participant-matched study, not as validated imaging biomarkers. This distinction is central for trustworthy AI in medicine, where apparent cross-dataset convergence can be misleading if acquisition, phenotype definition, and sampling frame differ (Norgeot et al., 2020).

The molecular rankings should be interpreted with similar restraint. Dopamine-related genes, lysosome-autophagy, mitochondrial biology, and neuroimmune modules are well described in prior PD research (Dehay et al., 2013; Bose and Beal, 2016; Hirsch and Hunot, 2009), and their appearance here indicates consistency with that literature. It does not show that any pathway mediated the PPMI association: the pathway modules were prespecified, candidate scores incorporated literature priors, tissues and sampling frames differed, and no participant had linked clinical, imaging, and molecular measurements. The digital datasets likewise show that wearable and voice measurements can capture movement, diagnosis, or UPDRS-related signals in their source tasks; they do not establish digital biomarkers of cognitive progression. Their value is limited to planning what might be collected prospectively alongside mood, cognition, and imaging.

The clinical implication is a testable monitoring strategy rather than an immediately deployable prediction rule. A brief baseline inventory covering depression, anxiety, sleepiness, autonomic burden, apathy, fatigue, and impulse-control symptoms could serve as a low-cost first-stage risk-enrichment measure. Patients with high burden could be considered for more structured follow-up, including informant history and domain-specific cognitive assessment in addition to global screening. The present data do not establish an optimal interval, but they motivate testing whether 6- to 12-month cognitive review, rather than symptom-triggered testing alone, improves timely detection in high-burden patients. Because the incremental predictive value and decision consequences have not been prospectively evaluated, the score should not currently determine diagnosis, treatment, or surveillance frequency by itself.

For clinical trials, the phenotype could be evaluated as an enrichment or stratification factor to increase the proportion of participants likely to show cognitive change, balance non-motor burden across randomized arms, and prespecify heterogeneity analyses. Trial use would require external calibration, assessment of selection bias, and demonstration that enrichment improves efficiency without excluding important low-burden progressors. The association also motivates intervention studies, but it must not be interpreted to mean that treating depression, anxiety, or sleepiness will necessarily prevent cognitive decline. Future trials should distinguish symptomatic benefit from disease-modifying effects and should measure both changes in non-motor burden and repeated, domain-specific cognition.

The methodological contribution is therefore a framework for future same-participant evaluation, not completed multimodal modeling. The current study establishes a clinically grounded follow-up association and uses separate datasets to specify testable imaging, molecular, and digital hypotheses. A future participant-matched design should collect repeated clinical assessments, harmonized imaging, digital measures, and biospecimens in the same individuals; prespecify missing-data handling and temporal alignment; replicate the clinical association; and evaluate calibration, fairness, incremental predictive value, and clinical utility before deployment. The advantage of starting with an interpretable phenotype is that later machine-learning models can be benchmarked against a transparent clinical signal rather than optimized around an opaque endpoint (Collins et al., 2015; Topol, 2019).

**Figure 7:**
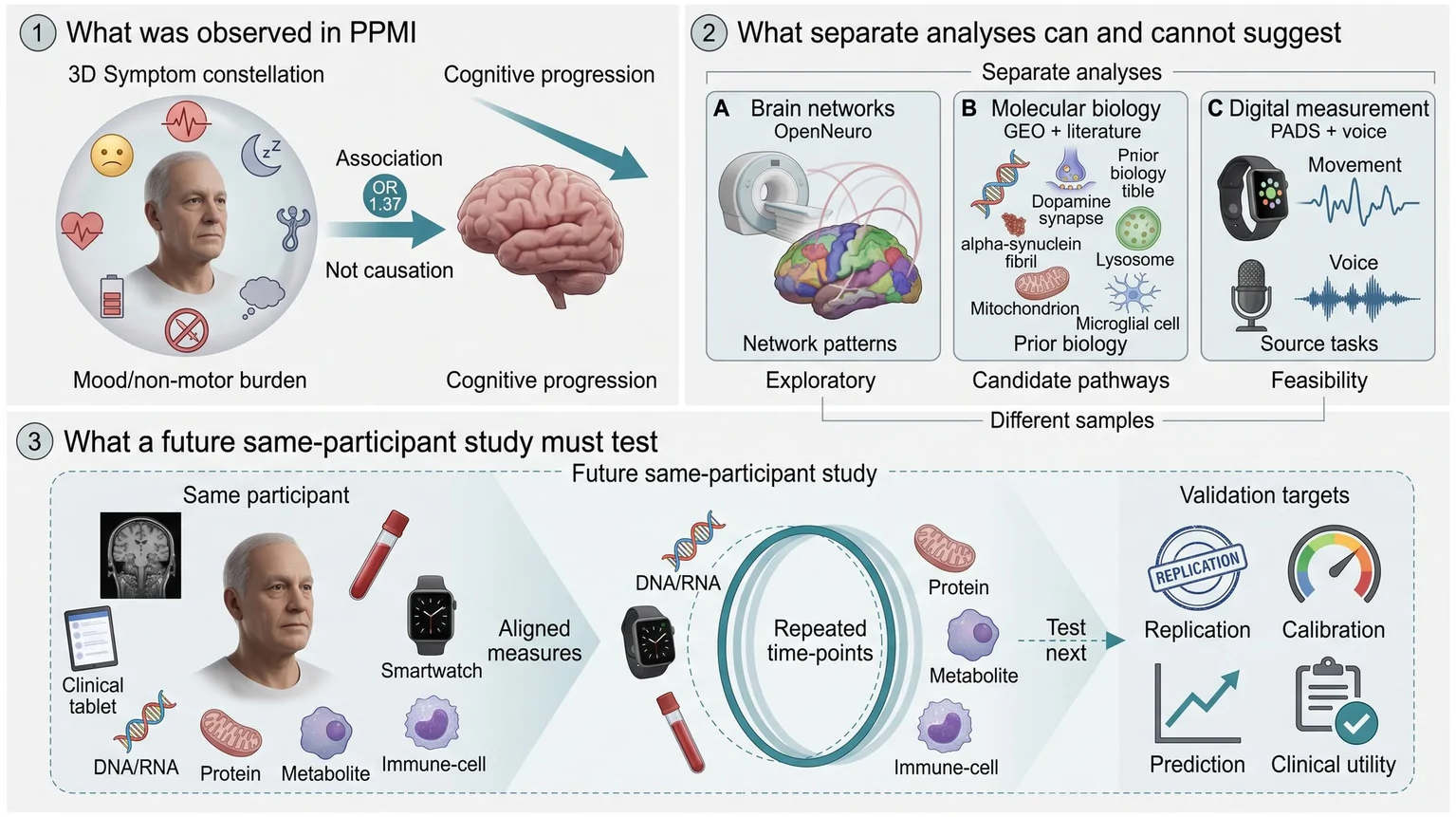
Observed clinical association, separate analyses, and future same-participant testing. In PPMI, higher baseline mood/non-motor burden was associated with cognitive progression (adjusted OR per SD 1.37); this association does not establish causation. Separate analyses using OpenNeuro, GEO and prior literature, and PADS and voice data addressed exploratory network patterns, candidate biological pathways, and measurement feasibility, respectively. These analyses involved different samples and did not explain or validate the PPMI association. A future study should align repeated clinical, imaging, digital, and molecular measurements within the same participants and evaluate replication, calibration, incremental predictive value, and clinical utility.

Several limitations constrain interpretation. The clinical outcome used a pragmatic windowed composite of cognitive-category worsening or MoCA decline rather than a time-to-event estimand; interval censoring and the increasing opportunity to observe events explain why event rates rose across longer windows. Although ORs were stable from 1 to 5 years, this sensitivity analysis does not remove informative loss to follow-up. The 173 participants without evaluable cognitive follow-up were excluded rather than treated as non-progressors, but selection bias, exposure and outcome measurement error, and residual confounding remain possible. Bootstrap and repeated cross-validation assess internal stability only; the small AUC increment and absence of external clinical validation are important limitations. The OpenNeuro fMRI cohort was small and external to PPMI; most imaging comparisons were not significant after correction; and it lacked aligned mood/non-motor measures. Molecular and digital resources used different tissues, outcomes, and sampling frames, while multi-omics scores were planning constructs. In conclusion, baseline mood/non-motor burden was associated with cognitive progression in PPMI and may be useful for prospective risk-enrichment research. Participant-matched studies are required to replicate the association and evaluate calibration, incremental predictive value, and clinical utility before any measure is used as a biomarker or decision tool (Figure 7).

## Data Availability

The raw data supporting the conclusions of this article will be made available by the authors, without undue reservation.

## Glossary

AI: Artificial intelligence
aCompCor: Anatomical component-based noise correction
AUC: Area under the receiver operating characteristic curve
BOLD: Blood-oxygen-level-dependent
CAP: Coactivation pattern
CI: Confidence interval
DTI: Diffusion tensor imaging
EEG: Electroencephalography
ESS: Epworth Sleepiness Scale
FDR: False discovery rate
fMRI: Functional magnetic resonance imaging
Fnirs: Functional near-infrared spectroscopy
FWE: Family-wise error
GABA: Gamma-aminobutyric acid
GBD: Global Burden of Disease
GDS: Geriatric Depression Scale
GEO: Gene Expression Omnibus
KEGG: Kyoto Encyclopedia of Genes and Genomes
LEDD: Levodopa-equivalent daily dose
MCI: Mild cognitive impairment
MDS: Movement Disorder Society
MDS-UPDRS: Movement Disorder Society-Unified Parkinson’s Disease Rating Scale
MEG: Magnetoencephalography
MNI: Montreal Neurological Institute
MoCA: Montreal Cognitive Assessment
NBS: Network-based statistic
OR: Odds ratio
PADS: Parkinson’s Disease Smartwatch dataset
PCA: Principal component analysis
PD: Parkinson’s disease
PD-MCI: Parkinson’s disease with mild cognitive impairment
PD-NC: Parkinson’s disease with normal cognition
PPMI: Parkinson’s Progression Markers Initiative
PRIMDIAG: PPMI primary-diagnosis variable
QUIP: Questionnaire for Impulsive-Compulsive Disorders in Parkinson’s Disease
RMS: Root mean square
ROI: Region of interest
SCOPA-AUT: Scales for Outcomes in Parkinson’s Disease-Autonomic
SD: Standard deviation
STAI: State–Trait Anxiety Inventory
TR: Repetition time
UCI: University of California Irvine
UPDRS: Unified Parkinson’s Disease Rating Scale
